# The Defense Metabolite, Allyl Glucosinolate, Modulates *Arabidopsis thaliana* Biomass Dependent upon the Endogenous Glucosinolate Pathway

**DOI:** 10.3389/fpls.2016.00774

**Published:** 2016-06-01

**Authors:** Marta Francisco, Bindu Joseph, Hart Caligagan, Baohua Li, Jason A. Corwin, Catherine Lin, Rachel Kerwin, Meike Burow, Daniel J. Kliebenstein

**Affiliations:** ^1^Department of Plant Sciences, University of CaliforniaDavis, CA, USA; ^2^Group of Genetics, Breeding and Biochemistry of Brassicas, Department of Plant Genetics, Misión Biológica de Galicia, Spanish Council for Scientific ResearchPontevedra, Spain; ^3^DynaMo Center of Excellence, Copenhagen Plant Science Centre, University of CopenhagenFrederiksberg, Denmark

**Keywords:** Arabidopsis, allyl GSL, GSL-induced responses, plant growth, defense metabolism

## Abstract

Glucosinolates (GSLs) play an important role in plants as direct mediators of biotic and abiotic stress responses. Recent work is beginning to show that the GSLs can also inducing complex defense and growth networks. However, the physiological significance of these GSL-induced responses and the molecular mechanisms by which GSLs are sensed and/or modulate these responses are not understood. To identify these potential mechanisms within the plant and how they may relate to the endogenous GSLs, we tested the regulatory effect of exogenous allyl GSL application on growth and defense metabolism across sample of *Arabidopsis thaliana* accessions. We found that application of exogenous allyl GSL had the ability to initiate changes in plant biomass and accumulation of defense metabolites that genetically varied across accessions. This growth effect was related to the allyl GSL side-chain structure. Utilizing this natural variation and mutants in genes within the GSL pathway we could show that the link between allyl GSL and altered growth responses are dependent upon the function of known genes controlling the aliphatic GSL pathway.

## Introduction

To ward off biotic attackers, plants utilize a wide array of physical defenses, such as trichomes and wax layers that deter access to the tissue. Another layer of defense is provided by a diversity of chemicals that affect the biotic attacker's physiology and reproductive potential (Levin, 1973; Feeny, 1976). These defenses are hypothesized to be costly to the plant, diverting energy and resources away from primary metabolic functions and toward generating these structures and compounds (Karban and Baldwin, 1997; Baldwin, 1998; Mauricio, 1998; Cipollini et al., 2003; Paul-Victor et al., 2010; Züst et al., 2011). As well as the potential metabolic cost, there are strong ecological costs that may be as strong or stronger than the metabolic cost, e.g., an effective defense against one organism may actually generate susceptibility against a different organism (Karban and Baldwin, 1997; Heil, 2002; Kliebenstein and Rowe, 2008). An example of this are the glucosinolate (GSL) defense metabolites in the order Brassicales that provide resistance to generalist herbivores and concurrently provide signals that attract and benefit infestation by specialist herbivores (Renwick and Chew, 1994; Griffiths et al., 2001; Lambrix et al., 2001; Kliebenstein et al., 2002; Ratzka et al., 2002; Renwick et al., 2006; Pfalz et al., 2007, 2009; Beekwilder et al., 2008; Hansen et al., 2008). Similarly ecological costs are being noted where resistance genes against one pathogen are beginning to be found as susceptibility genes against counter-adapted pathogens (Lorang et al., 2004, 2007). These countervailing costs and benefits of defense, requires that the plant properly calibrates its defense within any given environment to optimize the ratio of all benefits to all costs.

One method to dynamically balance the plant's defense portfolio is an integrated regulatory system that senses as many aspects of the biotic environment as possible. This system would then integrate these inputs from the biotic environment into the signaling network to properly modulate the defense outputs. This regulatory architecture involves a myriad of interconnections between the different pathways, allowing integration of different perception signals and generation of a coordinated output (Kunkel and Brooks, 2002; Kovac et al., 2009; Horstman et al., 2013; Zhang et al., 2013). The current model of plant defense signaling as a hierarchical regulatory system is, however, unlikely to properly explain the full complexity of plant defense networks. Current systems engineering theory suggests that purely hierarchical systems, like most plant network models, are unstable due to a lack of negative feedback and output integration (Kerwin et al., 2011; Pourcel et al., 2013; Bonawitz et al., 2014). Therefore, a truly stable system must have some form of feedback that connects system outputs to inputs to optimize effectiveness. Evidence that defense metabolites can provide this feedback regulation in plants is beginning to accumulate (Kerwin et al., 2011; Pourcel et al., 2013; Bonawitz et al., 2014). Recent work has suggested that plant specialized metabolites, such as GSLs and phenylpropanoids, have downstream regulatory influences on the plant in which they are synthesized (Kerwin et al., 2011; Pourcel et al., 2013; Bonawitz et al., 2014). In *Arabidopsis thaliana*, an indolic GSL compound alters defense signaling, suggesting that defense outputs can feedback modulate upstream regulatory processes. Similarly, in *Raphanus sativus*, a GSL hydrolysis product can directly modulate plant physiology by affecting the TIR1 auxin receptor (Hasegawa et al., 2000; Yamada et al., 2003; Clay et al., 2009). Genetic evidence also suggests that the aliphatic GSL pathway influences a myriad of other pathways. For example, a number of GSL mutants have significant growth defects, suggesting the potential for cross-talk between the GSL pathway and hormone metabolism (Delarue et al., 1998; Barlier et al., 2000; Hansen et al., 2001; Chen et al., 2003; Mikkelsen et al., 2004). More directly, the introduction of a functional AOP2, a biosynthetic enzyme in the aliphatic GSL pathway, into a naturally occurring AOP2 knockout genotype altered flowering, jasmonyl-isoleucine (JA-ILE) mediated defense signaling and oscillatory behavior of the circadian clock (Wentzell et al., 2007; Kerwin et al., 2011; Burow et al., 2015). The results indicate that at least the AOP2 RNA interconnects with cellular signaling and metabolic pathways via unknown mechanisms (Burow et al., 2015). However, it is unclear if GSL metabolites alone can modulate plant physiology. Thus, there is the potential that specific GSL metabolites, in contrast to the RNAs, may provide direct feedback regulation within the plant, but there is a need to test whether the metabolite controls these processes and to identify the genes and mechanisms that may facilitate this.

While the above efforts have started to show that GSLs can modulate regulatory pathways, our knowledge of the underpinning molecular mechanisms by which the GSL pathway is linked to other pathways in the plant, and how the connections translate into physiological output of plant growth, development, and defense is limited. To begin testing if a GSL metabolite produced by AOP2 can function as a feedback signal that alters plant biomass and defense metabolism, we fed exogenous allyl GSL (also known as 2-propenyl GSL or sinigrin) to *A. thaliana* and uncovered a wide range of heritable effects upon growth and endogenous GSL accumulation. Fifty micrometer allyl GSL was introduced exclusively to the roots and the compound was transported up to the leaf where it accumulated as less than 5% of the endogenous pool in any of the tested accessions. Using a population of 96 natural *A. thaliana* accessions and mutant genotypes in the GSL pathway we showed that exogenous allyl GSL has the capacity to differentially affect plant biomass and metabolite content of Arabidopsis dependent upon the environment and endogenous GSL genetic variation. Future work is required to test if allyl GSL or a related derivative is the active component and where the site(s) of activity are located.

## Materials and methods

### Plant material and exogenous allyl GSL feeding experiment

*Arabidopsis thaliana* seeds were surface-sterilized (1-min, 70% ethanol soaking followed by a 20-min, 50% sodium hypochlorite), rinsed (five times) in sterile, distilled water. They were then placed on petri dishes containing half-strength Murashige and Skoog (MS) salt medium (CAISSON, MSP01-1LT) adjusted to pH 5.8, containing 0.8% agar and 1% sucrose (control). To study the effect of exogenous allyl GSL on plant biomass and metabolite content, 0.22 μm filter sterilized allyl GSL 100 mM stock solution (Sigma S1647-1G) was added to the autoclaved MS (at 55°C) to a final concentration of 50 μM (treatment). To identify suitable genetic screening conditions, we initially tested different sucrose concentrations (0-1-2%) by planting 20 seeds in a 55 mm Petri dish from each of seven *A. thaliana* accessions (Bay-0, Col-0, Ler-0, Tsu-1, Cvi-1, Kas, and Sha) with three independent biological replications for each treatment. The entire above experiment was replicated in triplicate providing an N of 180 seedlings per accession per treatment. All plantings were independently randomized.

The study was increased to a survey of a 96 *A. thaliana* natural accessions (Nordborg et al., 2002, 2005; Borevitz et al., 2007; Atwell et al., 2010; Chan et al., 2010a,b, 2011; Table S1) and GSL genes mutant genotypes (*AOP2, gsm1, myb28/29, myb28, myb29, gsox1/3*, and *gsox3*; Table 1; Haughn et al., 1991; Kliebenstein et al., 2001a; Hansen et al., 2007; Sønderby et al., 2007, 2010; Li et al., 2008). For natural accessions, seeds were placed in 36 grid square 100 × 15 mm plates. Five plants per accession were grown in a randomized partial block design (one seed per grid square). Seeds were planted on control MS and allyl-containing MS to provide five measurements per accession per treatment. For GSL gene mutant genotypes, six plants per line were grown in a randomized partial block design (two seed per grid square and 10 blocks) providing 60 measurements per genotype per treatment. After planting on media, plates were stratified for 3 days in the dark at 4°C to break dormancy. Plates were then transferred to a growth chamber under long-day conditions (16 h light at 100–120 μEi, 20°C). Any seedlings with leaf contact to the agar were removed from the analysis to ensure that root-to-shoot transport had occurred. At 15 days post germination, the rosette of each seedling was harvested from the plates, weighed to record the plant fresh weight (fw), then placed into a 96-deep well tube containing 90% methanol for GSL extraction and analyzed for GSL content as described below.

**Table 1:** **Description of the single and double mutants on GSL genes**.

**GSL mutant gene**	**Mutant name**	**Gene ATG #**	**Phenotype**
MYB28	*myb28*	At5g61420	Reduced levels of aliphatic GSL
MYB29	*myb29*	AT5g07690	Reduced levels of aliphatic GSL
MYB28/MYB29	*myb28/29*	At5g61420/AT5g07690	Absence of aliphatic GSL
MAM1	*gsm1*	At5g23010	Accumulation of C3 GSL
GSOX1/GSOX3	*gsox1/3*	At1g65860/At1g62560	Accumulation of methylthioalkyl GSL
GSOX3	*gsox3*	At1g62560	Accumulation of methylthioalkyl GSL
AOP2	*AOP2*	At4g03060	Accumulation of alkenyl GSL

### Analysis of GSL content

GSLs of excised shoots were measured using a previously described high-throughput analytical system (Kliebenstein et al., 2001a,b,c). Briefly, rosettes of all seedlings were individually removed from plates with forceps, weighed and placed in a single well of 96-well microliter plate containing 400 μL of 90% methanol and one 3.8 mm stainless steel ball-bearing. Tissues were homogenized for 3 min in a paint shaker, centrifuged, and the supernatants transferred to a 96-well filter plate with 50 μL of DEAE sephadex and washed once with water. The sephadex-bound GSL were eluted by overnight, room temperature incubation with 110 μL of sulfatase. Individual desulfo-GSLs within each sample was separated and detected by HPLC-DAD, identified, and quantified by comparison to purified standards. The GSL traits are reported as μmol g of fw of each plant. All seedlings were measured individually and GSL abundance was normalized to the fresh weight. In addition to the content of individual GSLs, we developed a series of summation and ratio traits based on prior knowledge of the GSL pathways (Table S2; Kliebenstein, 2007; Wentzell et al., 2007).

### Quantitative real-time PCR

RNA from Col-0 genotype was extracted from three pools of three seedlings for each treatment (MS and MS + allyl) with Sigma Spectrum Plant Total RNA kit, treated with Sigma DNAse1 and reverse transcribed with iScript (Bio-rad). Expression was assayed by quantitative real-time PCR using SYBR Green and the data was normalized to *UBC* expression of each pool. The following primers were used: *UBC* (At5g25760), 5′-CTGAGCCGGACAGTCCTCTTAACTG-3′ and 5′-CGGCGAGGC GTGTATACATTTGTG-3′; *MYB28* (At5g 61420), 5′-TC CCCAAAAAGCTGGGTTGAAA-3′ and 5′-TTTA AGGTAGTTGGTCCATCGCA-3′; *MYB29* (At5g07690), 5′-GA ACACGCATCTCAAAAAGCTCCTG-3′ and 5′-ACTTTGGAG AGATGGAACCCGATTG-3′; *MAM3* (At5g23020), 5′-CG CTGATCTGAAGGCATTAGTGGTG-3′ and 5′-GCGGAAATCTGAGGGCTTGACATA-3′; *CYP83A1* (At4g13770), 5′-TCTCGCCGCGGTTCTCCTTT-3′ and 5′-GCCCATCCAGCGAAGAAGCGT-3′; *GS-OX1* (At1g65860), 5′-GCCGGTTAACGGGAAATGGAGTGT-3′ and 5′-ATTTCATGGGCGGCGAAACCAA-3′. Gene expression levels are presented as mean fold difference between treated and untreated as obtained across two independent experiments with three biological replicates per experiment.

### Statistical analyses

To test how the plant biomass and GSL responses to allyl treatment interact with the growth media, we conducted a three-way ANOVA using the factors accession, allyl treatment (MS and MS + allyl), MS media (0, 1, and 2% Sucrose) as well as the interactions between these factors. Plate was tested for significance as a random effect in a mixed model but not found to significantly alter the results and hence dropped from the model. The least-square means of each plant biomass and GSL phenotype per each accession within each condition were obtained using this model. ANOVA was also utilized to test for the effect of exogenous allyl GSL on plant biomass and GSL content of different GSL gene mutant lines. Each mutant was tested in an individual ANOVA against the wild-type (WT) Col-0 genotype. Multiple comparisons were made *post-hoc* using Tukey's *t*-test with *P* ≤ 0.05 within the model.

To directly test if the GSL profile of the different accessions was influencing the response to exogenous allyl GSL, we utilized the mean phenotypes for each accession within each treatment. Using their GSL chemotype, we assigned each accession their appropriate genotype at the *GS-AOP* (methylsulfinyl, AOP1; alkenyl, AOP2 and hydroxyalkyl, AOP3) and *GS-Elong* (3C vs. 4C) loci. None of the studied accessions displayed the AOP3 C4 chemotype. We then conducted an ANOVA using the *GS-AOP* genotype (AOP1, AOP2, and AOP3), *GS-Elong* genotype (3C and 4C) and allyl treatment as factors. We also explicitly tested the various interaction of these factors in the model; *GS-AOP* × *GS-Elong, GS-AOP* × allyl treatment, *GS-Elong* × allyl treatment and *GS-AOP* × *GS-ELong* × allyl treatment.

To test for correlations in the concentrations of individual GSL compounds or classes to the changes in plant biomass we used Spearman's correlation test within a stepwise linear regression model. For these analyses, we tested for a correlation of the relative plant biomass response with the individual relative GSL responses. All the relative responses for each trait were calculated as: $\frac{\left(\text{MS }+\text{ Allyl}\right)\;-\;\left(\text{MS}\right)}{\frac{1}{2}\left[\left(\text{MS }+\text{ Allyl}\right)\;+\;\left(\text{MS}\right)\right]}$ (MS stands for MS media with (MS+allyl) or without (MS) exogenous allyl). For the stepwise model, only the variables with a *P* = 0.5 for entry and *P* = 0.1 for removal were kept in the model. All statistical analyses were conducted using SAS.

## Results

### Allyl GSL fed plant biomass under different sucrose concentration

**Figure 1:**
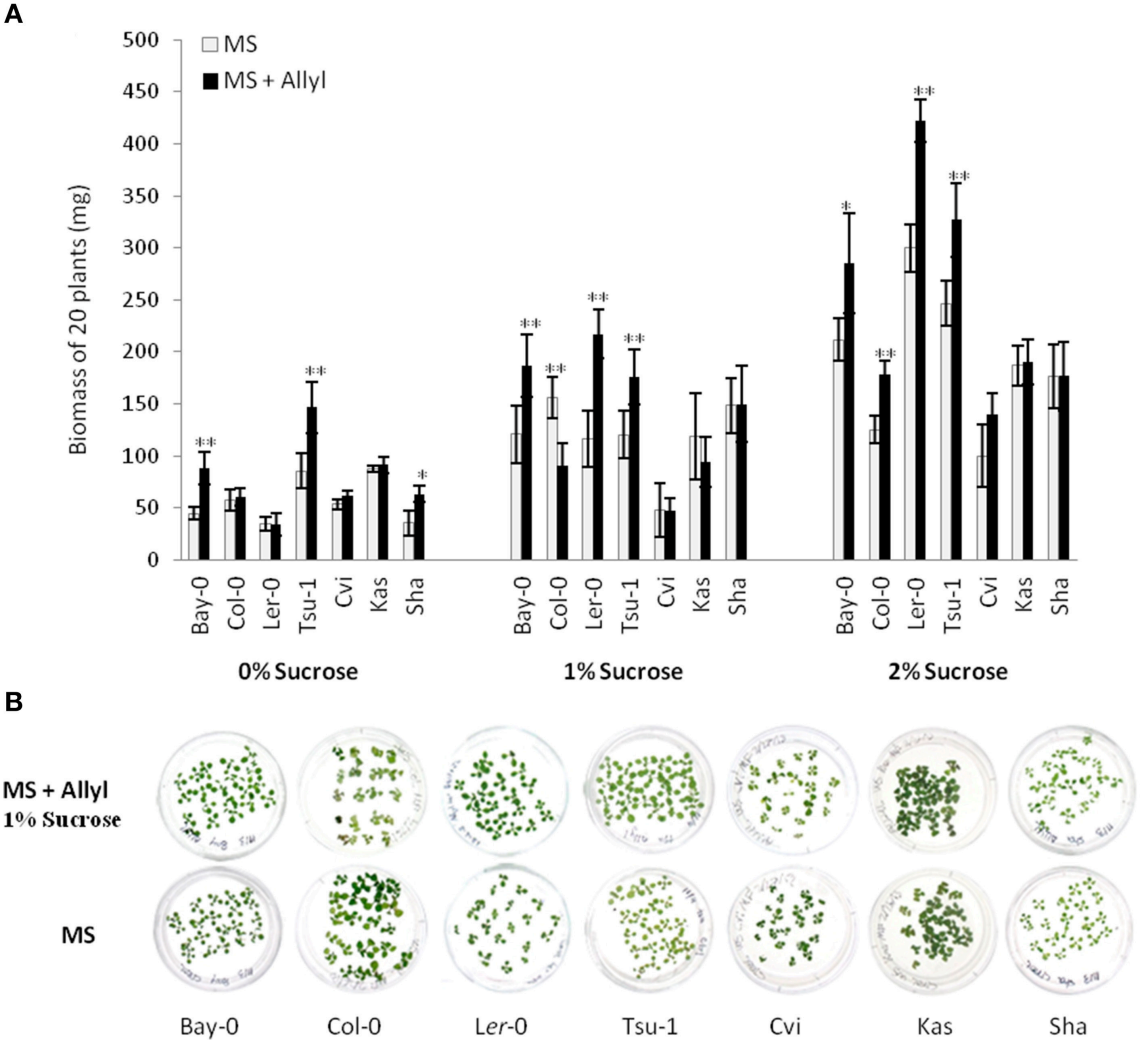
**Differential plant biomass responses of Arabidopsis accessions to exogenous allyl GSL is sugar dependent. (A)** Quantification of biomass of 15-day-old seedlings from seven *A. thaliana natural* accessions fed with 50 μM of allyl GSL using MS media differing in sucrose concentrations. The accessions Bay-0, Col-0, L*er*-0, and Tsu-1 do not synthesize endogenous allyl GSL, while the accessions Cvi, Kas, and Sha produce endogenous allyl GSL. Asterisks indicates significant effect of the exogenous allyl GSL treatment on that accession under that condition, ^*^P ≤ 0.05 and ^**^P ≤ 0.01 from the ANOVA analysis (Table S3). The error bars represent standard deviation from three independent experiments with each bar showing the average biomass of at least 60 individual plants across these experiments. **(B)** Representative photographs of seven *Arabidoposis* accessions feed with or without 50 μM of allyl GSL at 1% of sucrose concentration.

Previous work has suggested that Arabidopsis and Raphanus GSLs can influence plant physiology (Hasegawa et al., 2000; Yamada et al., 2003; Kerwin et al., 2011). Thus, we proceeded to test if the allyl GSL that is produced in numerous Brassica species, including many but not all natural Arabidopsis accessions, could influence plant development. Because sucrose is well known to condition many growth phenotypes by influencing various regulatory processes, we grew Arabidopsis seedlings in MS media containing 50 μM allyl GSL with different sucrose concentrations (0, 1, and 2%) to identify optimal conditions for testing any observed responses (Figure 1, Table S3). This concentration of allyl GSL is approximately the endogenous concentration found in allyl GSL producing Arabidopsis accessions (Kliebenstein et al., 2001a,b,c; Wentzell et al., 2007; Chan et al., 2010b). To simultaneously test if any observed effects differed across accessions, we used seven Arabidopsis accessions that differ in their endogenous GSL profile. We measured the biomass of at least 180 individual seedlings at 15 days post-germination for each accession × sucrose × treatment combination spread across three independent experiments.

The addition of allyl GSL within the media led to increased biomass of most accessions, which was conditional upon the sucrose concentration with increasing effects as sucrose increased (Figure 1). In general, the accessions that do not synthesize endogenous allyl GSL had the largest response to exogenous allyl GSL application (Bay-0, Tsu-1, L*er*-0, and Col-0). Addition of allyl GSL to the media increased the biomass of Bay-0 and Tsu-1 at all sucrose concentrations and L*er*-0 at 1 and 2% sucrose. These three accessions produce predominantly 3-hydroxypropyl (3OHP) GSL. Another non-allyl GSL producing accession, Col-0, which produces primarily 4-methylsulfinylbutyl (4MSB), responded differently to addition of allyl GSL and sucrose, with growth being promoted at 2% sucrose but inhibited at 1%. In contrast, accessions that can synthesize allyl GSL, such as Cvi and Kas, showed no significant plant biomass response to exogenous allyl GSL feeding. The exception to this was Sha, which responded with increased biomass upon exogenous allyl application, but only when sucrose was not present in the media. Together this shows that allyl GSL has the capacity to affect plant biomass of Arabidopsis accessions in a manner that is dependent upon the growing conditions and endogenous GSL synthesis capacity.

### Allyl GSL effects are related to the side-chain structure

**Figure 2:**
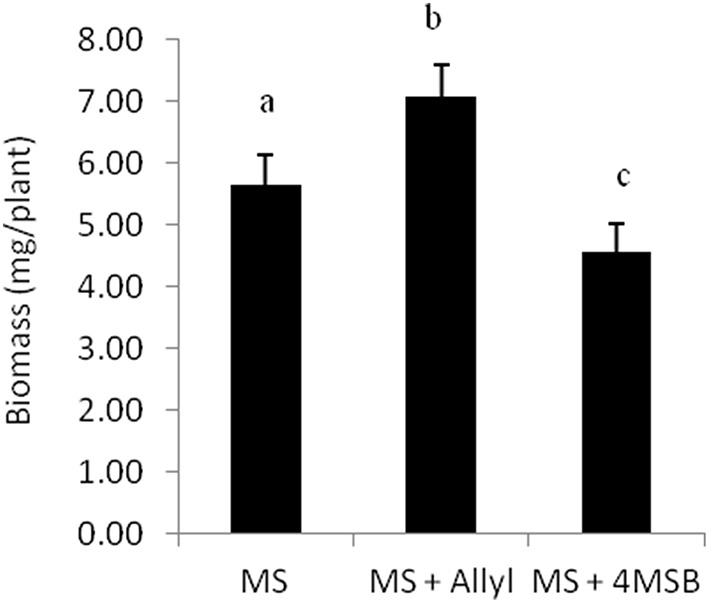
**Differential plant biomass responses of L*er*-0 accession fed with 50 μM of allyl and 4MSB GSLs using MS media at 1% of sucrose concentration**. The bar chart represents the mean fw (mg tissue/plant) and the error bars represent the standard deviation within each treatment (MS + allyl GSL; MS + 4MSB GSL). Letters indicate significant differences (*P* ≤ 0.05) between treatments using ANOVA and *post-hoc t*-test.

To assess if plant biomass responses are solely dependent on the core sulfate/thioglucose structure of generic GSLs or is related to the side-chain of allyl GSL, we tested the growth effect of 4MSB upon the L*er*-0 accession. For this test, we choose L*er*-0 since this accession showed the largest change in plant biomass when it was feed with allyl GSL. Moreover, L*er*-0 does not have the enzymes necessary to synthesize allyl or 4MSB GSL. Thus, L*er*-0 is naïve to both GSLs and any plant responses to these GSL could be directly interpreted in comparison to the negative control. Using 1% sucrose that maximized the L*er-*0 response to allyl GSL confirmed that allyl GSL stimulated biomass accumulation in these conditions (Figure 2). In contrast, 4MSB GSL lead to a significant decrease in biomass accumulation in Ler-0 under these conditions. As both GSLs have glucose and sulfate as a core component of their structure, this argues against these two GSLs have different affects and as such, these effects are not solely determined by the common sulfate/thioglucose structure that shared by the two GSLs. The fact that allyl GSL and 4MSB GSL produce opposing effects on L*er*-0 biomass suggests that they are perceived via different mechanisms that are related to the differences in the two GSLs side-chains.

### Exogenous allyl GSL is taken up by the plant

Previous studies have shown that exogenously applied GSL can be transported and accumulate within the plant (Brudnell et al., 1999; Iqbal and Mollers, 2003; Hansen et al., 2008; Andersen et al., 2013). To test if the exogenous allyl GSL can be taken up by the plant roots and accumulate in the leaves, we measured the GSL levels within the above Arabidopsis accessions grown in the presence and absence of exogenous allyl GSL on MS with 0, 1, and 2% sucrose. Individual plants for these assays were chosen to ensure that there was no leaf contact with the media and any detected allyl GSL had to be taken up by the roots and transported to the leaves. The seedlings were measured individually for GSL accumulation and not pooled.

**Figure 3:**
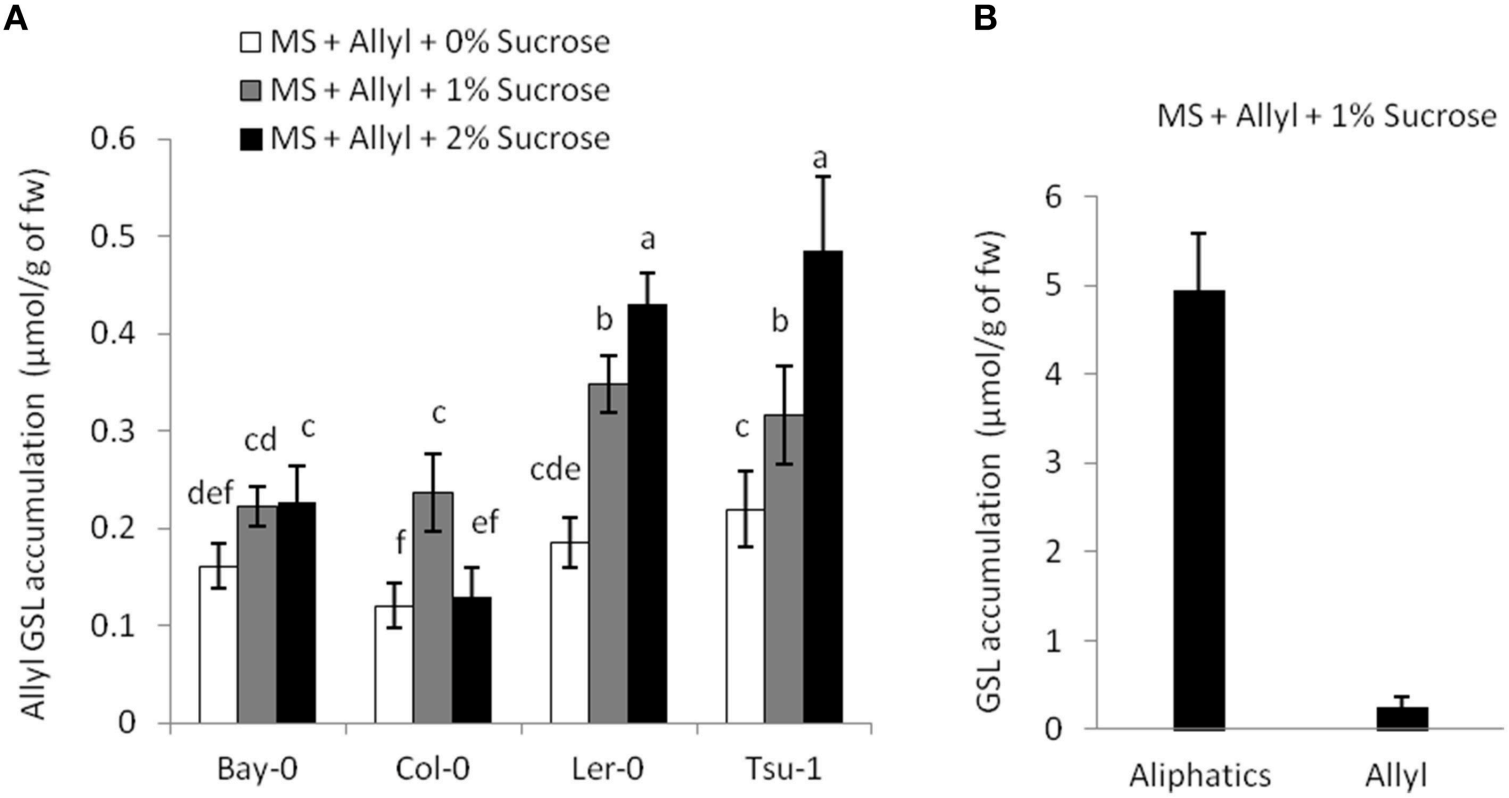
**Allyl GSL accumulation. (A)** The average of exogenous allyl GSL accumulation within leaves of 15-day-old seedlings was directly measured using HPLC. Shown are the accessions that are genetically incapable of synthesizing endogenous allyl GSL. Thus, detected allyl must have been taken up from the surrounding media, transported via the vasculature to the leaf and provides a direct measurement of the accumulation. Averages with the same letter are not significantly different at *P* ≤ 0.05 using a Tukey's *post hoc t*-test within the ANOVA. The error bars represent standard deviation from three independent experiments where 12 plants were separately measured per experiment. **(B)** Average of the total endogenous aliphatic GSL and accumulated allyl content within the accessions that do not synthesize endogenous allyl GSL grown in MS with allyl at 1% of sucrose on the growing media. The error bars represent standard deviation across accessions and experiments as described in part **(A)**.

The four Arabidopsis accessions within our experiment that do not produce endogenous allyl GSL are missing the necessary enzyme to make this compound. They either contain a five basepair frameshift deletion abolishing the protein or have a local inversion that removes the AOP2 promoter abolishing expression (Kliebenstein et al., 2001a; Chan et al., 2011). Therefore, in these accessions, all measured allyl GSL in the leaves of these seedlings must have been obtained from the exogenous feeding and provide a test for allyl GSL uptake and foliar accumulation. These accessions were able to take up allyl GSL from the media and this overall accumulation was dependent upon the sucrose concentration (Figure 3A). In general, allyl GSL accumulation increases with increasing sucrose content in all accessions, except for Col-0, which reached the highest allyl GSL foliar accumulation at 1% sucrose (Figure 3A). Interestingly, the highest variation in foliar accumulation of the exogenous allyl GSL across the accessions occurred with sucrose at 1%. At these conditions, the mean foliar accumulation of allyl GSL within accessions that cannot make allyl GSL was 0.28 μmol/g of fw. This concentration of exogenous allyl GSL was less than 5% of the total aliphatic GSL within these accessions (Figure 3B), showing that these treatment conditions have not artificially flooded the system. Further, this suggests that the lack of a plant biomass response in the allyl GSL producing accessions may be because they have sufficient internal allyl GSL such that the exogenous amount does not stimulate an additional response (Figure 1).

### Exogenous allyl GSL effects endogenous GSL accumulation

**Figure 4:**
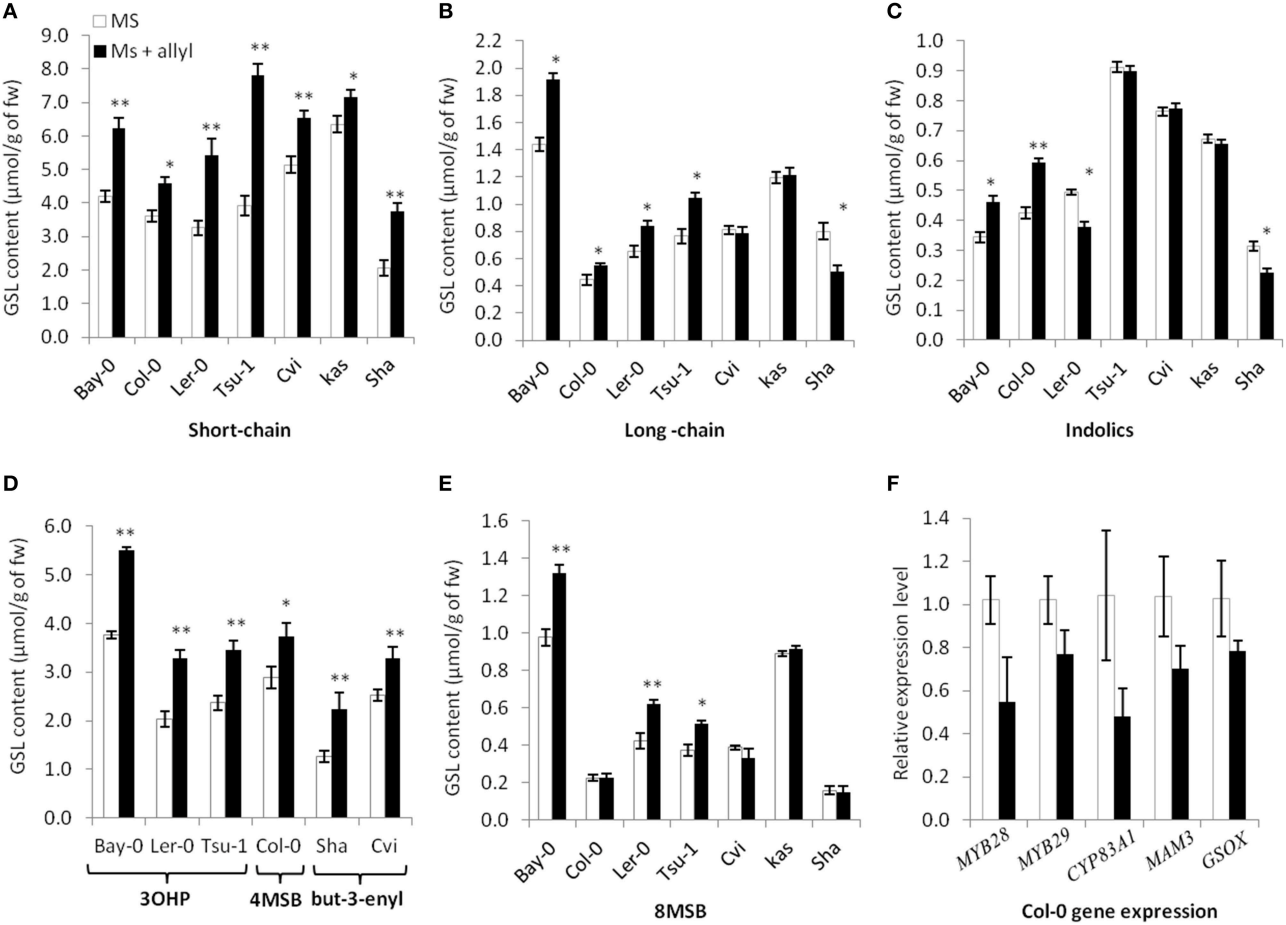
**Differential GSL responses of Arabidopsis accessions to exogenous allyl GSL**. Quantification of GSLs in 15-day-old seedlings from seven *A. thaliana natural* accessions fed with 50 μM of allyl GSL using MS media with 1% sucrose. Asterisks indicate a significant effect of the exogenous allyl GSL treatment on the accumulation of the shown GSL within the specific accession using a *post-hoc* Tukey test within the ANOVA analysis (^*^P ≤ 0.05 and ^**^P ≤ 0.01). The error bars represent standard deviation from three independent experiments with each bar showing the average biomass of at least 12 individual plants across these experiments. The GSL are as follows **(A**) Short-chain GSL content. **(B)** Long-chain GSL content. **(C)** Indolic GSL content. **(D)** Accumulation of the dominant short-chain GSL within each accession as shown by 3-hydroxypropyl (3OHP), 4-methylsulfinylbutyl (4MSB) and but-3-enyl GSL. **(E)** 8-methylsulfinyloctyl GSL (8 MSB) content. **(F)** qRT-PCR expression levels of five selected GSL biosynthetic genes in seedlings of Col-0 grown in the presence and absence of exogenous 50 μM of allyl GSL at 1% sucrose. The error bars represent standard deviation from two independent experiments with three biological replicates per experiment. None of the differences were statistically significant (ANOVA, *P* ≤ 0.05).

To test endogenous GSL levels may also respond to exogenous allyl GSL application, we measured GSL content from the seven Arabidopsis accessions seedlings (Bay-0, Tsu-1, L*er*-0, Col-0, Kas, Sha, and Cvi) fed with allyl GSL as well as from the control samples. These were from the same individual seedlings measured for biomass and all values are adjusted to the seedlings' biomass. These analyses detected 14 aliphatic GSL compounds and three indolic GSL traits. Since the accessions have different GSL profiles we focused on three GSL phenotypes that are measurable in all evaluated accessions (short-chain GSL accumulation, long-chain GSL accumulation, and indolic GSL accumulation). The accumulation of short-chain, long-chain and indolic GSLs showed statistically different responses to exogenous allyl GSL treatment across the accessions and sucrose concentrations (Table S4). The largest effect of exogenous allyl GSL on endogenous GSL accumulation was identified at 1% of sucrose (Figure 4). At these conditions, changes in endogenous GSL accumulation were larger than could be accounted for by the additive effect of exogenous GSL application and also affected a range of GSLs that cannot be synthesized from the allyl GSL, such as but-3-enyl GSL, 3OHP, 4MSB, and 8-methylsulfinyloctyl GSL (8 MSO) (Figure 4). Interestingly, while all the short-chain GSL positively responded to exogenous allyl GSL treatment in all accessions, the Cvi and Kas accessions had no long-chain GSL response to allyl GSL treatment. More dramatic was the indolic GSL accumulation where allyl GSL treatment induced the Bay and Col-0 accessions while repressing accumulation in L*er*-0 and Sha. Thus, exogenous allyl GSL causes diverse responses in the accumulation of endogenous GSL accumulation even in compounds that have little to no biosynthetic relationship (Figure 4).

### Understanding relationships between GSL content and plant biomass responses to exogenous allyl GSL

**Figure 5:**
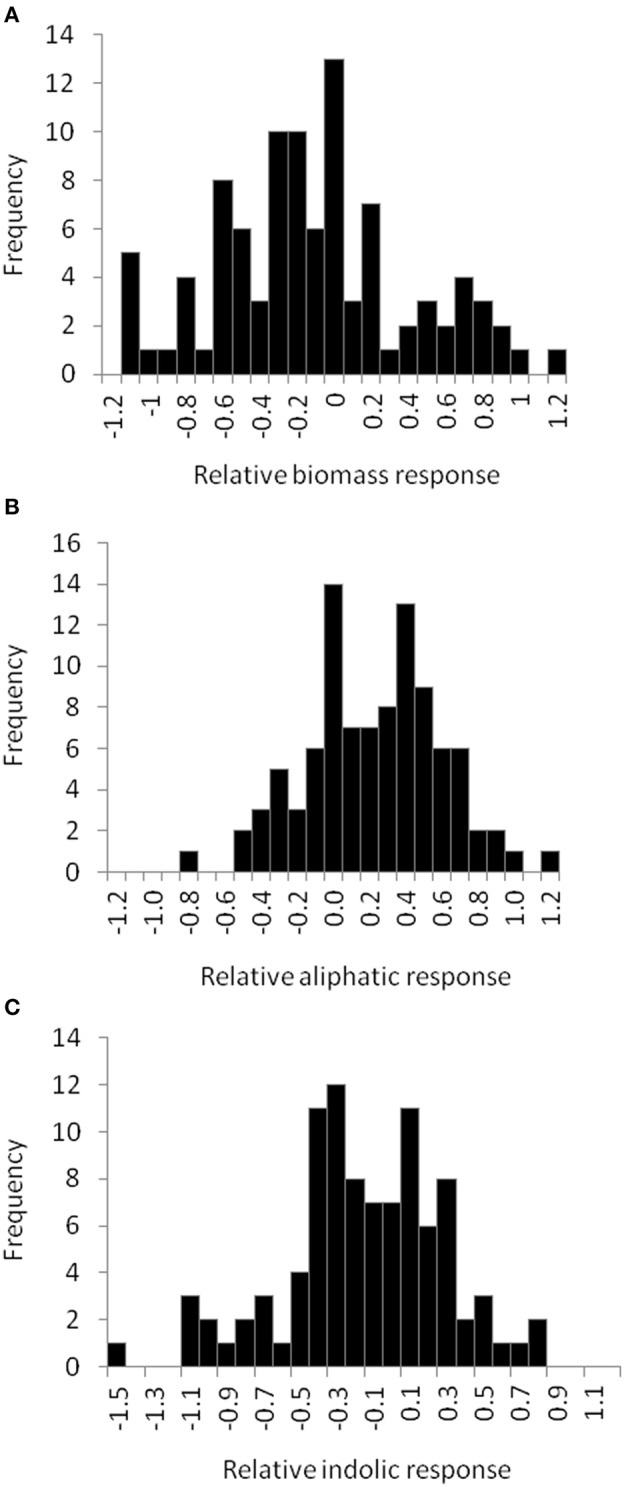
**Natural variation in Arabidopsis biomass and GSL acumulation in response to exogenous allyl GSL**. Histograms showing the frequency distribution of the relative responses to exogenous allyl GSL (50 μM of allyl GSL using MS media with 1% sucrose) across the 96 Arabidopsis natural accessions for the following phenotypes **(A)** plant biomass **(B)** total aliphatic GSL **(C)** total indolic GSL. The relative response of each phenotype to the allyl treatment within each accession was determined as (Phenotype in plants treated with allyl—phenotype in plants from the control treatement)/((0.5 × [Phenotype in plants treated with allyl + phenotype in plants from the control treatement]).

To begin identifying the potential mechanism(s) by which allyl GSL can stimulate changes in Arabidopsis biomass and defense, we expanded the study to a population of 96 natural Arabidopsis accessions. To maximize the potential phenotypic variance, we used the 1% sucrose concentration where we could identify both positive and negative effects on plant biomass and indolic GSL accumulation. The distribution of fresh weight across the accessions showed that individual accessions displayed both positive and negative changes in biomass in response to exogenous allyl GSL (Figure 5; Table S5). Thus, there is genetic variation for the plant biomass response to exogenous allyl GSL application in *A. thaliana*. GSL analysis detected 14 aliphatic GSL compounds and three indolic GSL compounds from which we focused on the traits described above (Wentzell et al., 2007; Chan et al., 2010b). As previously observed, exogenous allyl GSL treatment also altered endogenous GSL accumulation with varying effects across the Arabidopsis accessions (Figure 5, Table S5).

**Figure 6:**
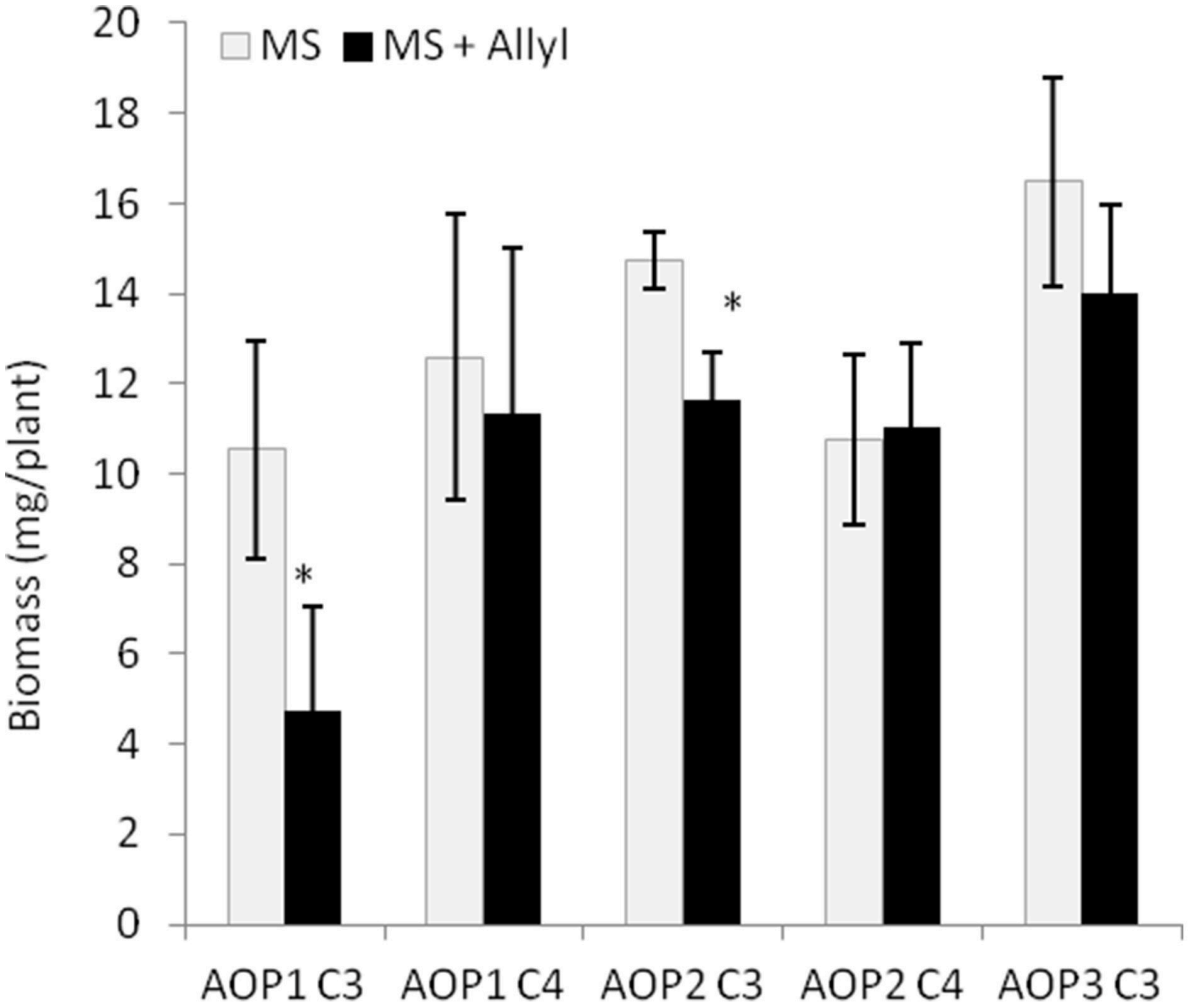
**GSL chemotype interacts with exogenous Allyl treatment to influence plant biomass of *Arabidopsis* accessions**. Quantification of fw (mg tissue/plant) from 15-day-old seedlings from 96 *Arabidopsis* natural accessions fed with 50 μM of allyl GSL using MS media with 1% sucrose. The population was grouped by their GSL chemotype profile generated by the combination of variation at *GS-AOP* (alkenyl, hydroxyalkyl, and methylsulfinyl) *GS-Elong* (3C vs. 4C). The sub-populations generated are labeled by their predominant glucosinolate; AOP1 C3 accumulates 3-methylsulfinylpropyl, AOP1 C4 accumulates 4-methylsulfinylbutyl, AOP2 C3 accumulates allyl. AOP2 C4 accumulates But-3-enyl and AOP3 C3 accumulates 3-hydroxypropyl (Table S1). None of the studied accessions displayed the AOP3 C4 chemotype accumulating 4-hydroxybutyl. The bar chart represents the mean fw and the error bars represent the standard deviation among the accessions within each chemotype. ^*^shows GSL haplotypes that had a significant difference in plant biomass response to exogenous allyl GSL using *post hoc* Tukey's *t*-test with *P* ≤ 0.05 from the ANOVA analysis.

The 96 Arabidopsis accessions have known and validated polymorphisms within key GSL loci allowing us to test if genetic variation in GSL loci altered the response to exogenous allyl GSL. For this analysis, we conducted an ANOVA using the GSL functional haplotype at the two major chemotype loci across the accessions as factors in the linear model. All accessions were assigned their functional haplotype at the *GS-Elong* locus, controlling the production of GSL with three carbon (C3) or four carbon (C4) side chains and *GS-AOP*, controlling the side-chain structure via three alleles AOP1, the null methylsulfinylalkyl allele; AOP2, the alkenyl allele and AOP3 the hydroxypropyl allele (Kliebenstein et al., 2001a,c). The ANOVA using the accessions showed that plant biomass varies significantly among treatments and GSL genotypes (Table 2). Further, there was a significant interaction of *GS-Elong* with allyl GSL treatment. In general, allyl GSL treatment had a stronger effect on plant biomass accumulation in genotypes which predominantly accumulate C3 GSL than those with C4 GSL (Figure 6). Thus, it appears that there is an association of the ability of the exogenous allyl GSL to affect plant biomass with the endogenous GSL genetic variation. However, the normal distribution of plant biomass responses across this Arabidopsis collection of accessions, with positive and negative responses (Figure 5), suggests that any underlying mechanism(s) are highly pleiotropic. Further, the variation of response within each chemotype shows that there are genes varying independently of the GSL loci that determine the final phenotypic response in each accession.

**Table 2:** **Effect of GSL haplotype upon the interaction of exogenous allyl treatment and plant biomass**.

**Source**	**Degrees of freedom**	**Sums of squares**	***F*-Value**	***P***
*GS-AOP*	2	2.4E-03	19.5	< 0.0001
*GS-Elong*	1	2.0E-05	3.3	0.0788
Allyl Treatment	1	4.4E-04	7.3	0.0107
*GS-AOP* × *GS-Elong*	1	1.1E-03	18.9	0.0001
*GS-AOP* × Allyl Treatment	2	1.2E-05	1.0	0.3767
*GS-Elong* × Allyl Treatment	1	2.5E-04	4.1	0.0494
*GS-AOP* × *GS-Elong* × Allyl Treatment	1	5.3E-06	0.1	0.7683
Block	4	1.3E-04	0.5	0.7050
Error	913	7.6E-02		

ANOVA was used to test the effect of the GSL haplotype within the 96 Arabidopsis natural accessions upon the biomass response to 50 μM of exogenously applied allyl GSL. The accessions were given their GSL genotypes at the two major structural loci, GS-AOP (alkenyl, hydroxyalkyl, and methylsulfinyl) and GS-Elong locus (3C vs. 4C). These were then treated as factors within the ANOVA. Type III Sums-of-squares are presented.

To extend this analysis to include all of the measure GSL, we tested for correlations between relative plant biomass response and the individual relative GSL traits responses to exogenous allyl GSL treatment. We found significant correlations between plant biomass response and 15 GSL traits. Among them, the strongest correlations were between plant biomass response and total aliphatic GSL accumulation (*R*^2^ = 0.49, *P* ≤ 0.001) and the ratio of methylthioalkyl/methylsulfinylalkyl GSL (*R*^2^ = −0.50, *P* ≤ 0.001). Both the methylthioalkyl and methylsulfinyalkyl GSL are upstream of allyl GSL in the aliphatic GSL pathway indicating a key role for early components of the GSL pathway in the response to the allyl GSL treatment (Table S6).

**Figure 7:**
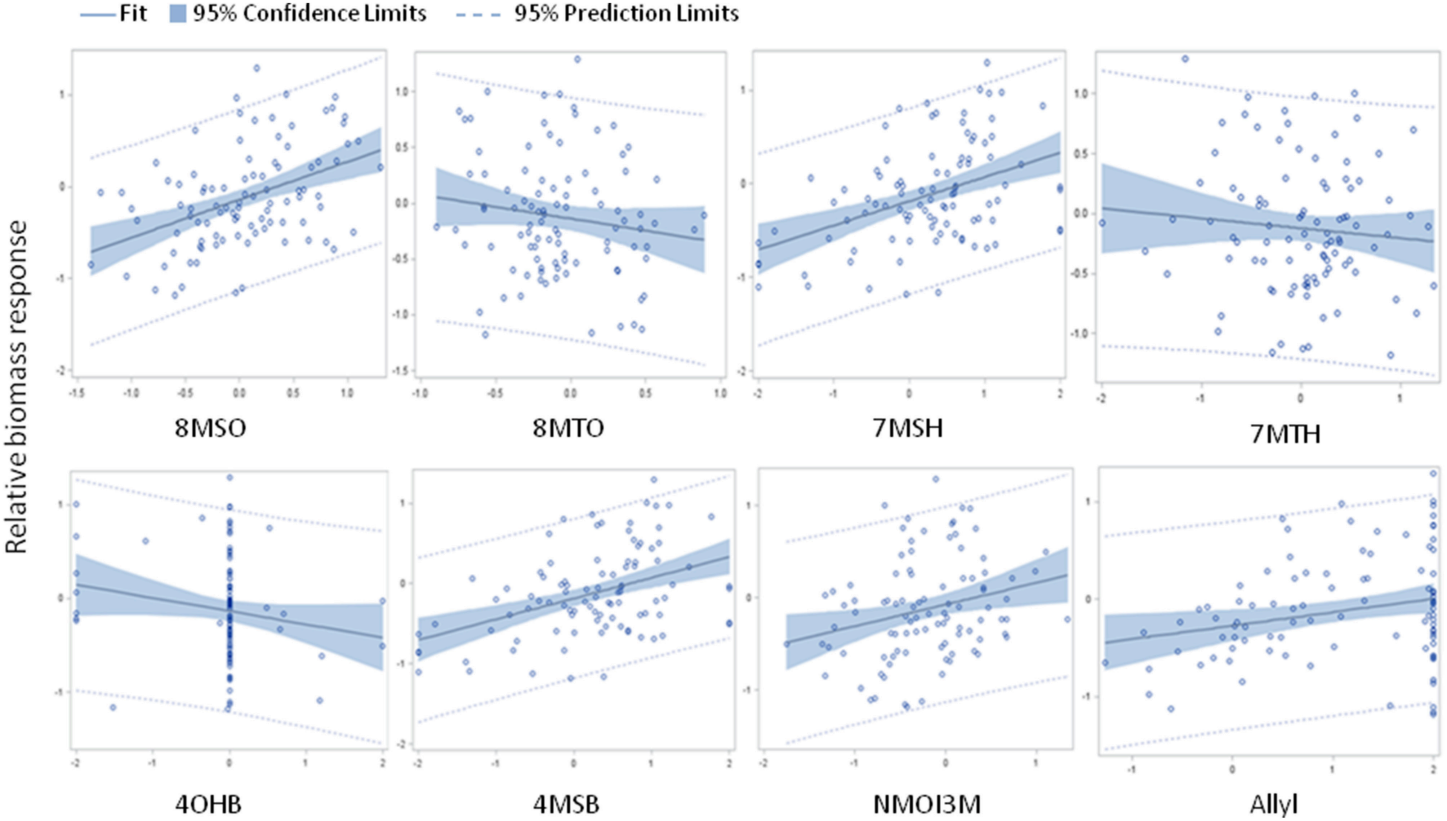
**Model results comparing relative plant biomass response to relative GSL content response to exogenous allyl GSL treatment**. Scatter plots with 95% confidence intervals on the partial correlations between relative plant biomass response (y axis) and the relative GSL content differences (x axis) for traits showing significance in the stepwise linear regression analysis (Table 3). See Table S2 for abbreviations.

The above analysis suggests that there may be multiple links between endogenous GSL variation and the response to exogenous allyl GSL with regards to plant biomass accumulation. To create a model linking how the different GSL compounds may combine to influence plant biomass responses to ally GSL, we used a stepwise regression analysis testing all the different GSL traits. This generated a model where variation in the response of eight GSL traits, seven aliphatics and one indolic, explained 43% of the variability in plant biomass response to exogenous allyl GSL across the accessions (Table 3). 8MSO was the compound with the strongest influence on plant biomass (*R*^2^ = 0.19) followed by 8-methylthiooctyl (8MTO) (*R*^2^ = 0.06). The other GSL traits, 4-hydroxybutyl (4OHB), 7-methylsulfinylheptyl (7MSH), allyl, 7-methylthioheptyl (7MTH), 4MSB, and N-methoxy-indol-3-ylmethyl (NMI3M) explained small but significant proportions of overall variance (Table 3). Interestingly, the correlation of plant biomass response with allyl GSL response was due to differences in endogenous allyl GSL content across the accessions that carried a functional AOP2 enzyme and is not a response to differential accumulation of exogenous allyl GSL. In concordance with our correlation analysis, the relationships between the two long chain methylthioalkyl GSL (8MTO and 7MTH) and plant biomass responses were negative, while the other traits were positively correlated (Figure 7). This suggests that variation in GSLs across the accessions is playing a role in the plant biomass response to exogenous allyl GSL treatment.

**Table 3:** **Stepwise regression model linking differential GSL and plant biomass responses to exogenous allyl GSL treatment**.

**GSL**	**Parameter estimate**	**Standard error**	**Partial *R*^2^**	**Mode *R*^2^**	***F***	**Pr > F**
8MSO	0.31	0.10	0.19	0.19	9.0	0.0036
8MTO	−0.30	0.13	0.06	0.25	5.3	0.0232
4OHP	−0.18	0.07	0.04	0.29	7.2	0.0090
7MSH	0.14	0.06	0.04	0.34	4.6	0.0358
Allyl	0.11	0.05	0.03	0.37	5.2	0.0248
7MTH	−0.19	0.08	0.02	0.39	5.3	0.0241
4MSB	0.10	0.05	0.02	0.41	3.7	0.0575
MI3M	0.15	0.09	0.02	0.43	3.3	0.0733
Intercept	−0.29	0.07			16.1	0.0001

Shown are the GSL selected as significantly correlated to differential plant biomass accumulation in response to exogenous allyl GSL across the accessions. The GSL variables are listed in order of their predictive strength with their individual contribution and total model R^2^ as well as significance within the combined model. See Table S2 for abbreviations.

The above effects of exogenous allyl on endogenous GSL accumulation suggest that there may be a transcriptional effect on the genes responsible for GSL biosynthesis. To test this hypothesis, we used qRT-PCR to measure the expression of five aliphatic GSL related genes (*MYB28, MYB29, CYP83A1, MAM3*, and *GS-OX1*). Using two independent experiments and three biological replicates per experiment, we harvested Col-0 leaves grown in the presence and absence of exogenous allyl GSL and measured transcript accumulation for the above genes by qRT-PCR. Analysis of this data identified no statistically significant differences between the treated and untreated samples for any genes (ANOVA, *P* > 0.05; Figure 4F). As such, there is no statistical support to argue that allyl GSL leads to direct alterations in transcript accumulation for aliphatic GSL genes. There are other reported instances where transcript abundance and metabolite abundance do not correlate within the aliphatic GSLs. For example, single knockouts in *MYB28* and *MYB29* generate the same reduction in metabolite accumulation but have disparate effects on transcript abundance (Sønderby et al., 2007, 2010). This suggests that whole genome transcriptomics will be required to test for any transcriptional effects of allyl GSL application.

### Evaluation of known GSL mutant genotypes treated with exogenous allyl GSL

**Figure 8:**
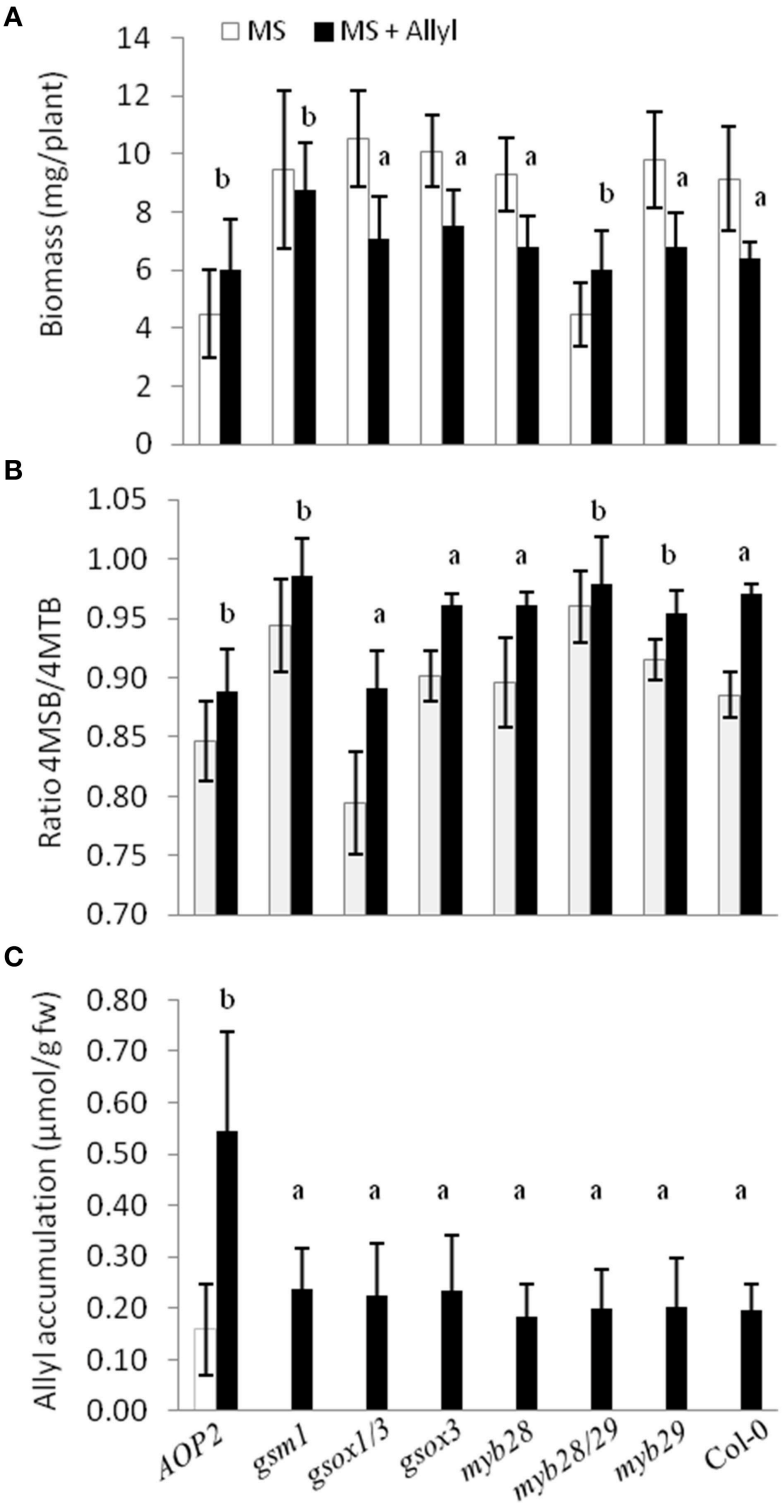
**Plant biomass responses and GSL content variation among GSL mutant genotypes treated with exogenously applied allyl GSL. (A)** Quantification of 15-day-old fw (mg/plant) seedlings from seven GSL mutant genotypes and wild-type (Col-0) fed with 50 μM of allyl glucosinolate using MS media with 1% sucrose. **(B)** Ratio of 4-methylsulfinylbutyl (4MSB)/4-methylthiobutyl (4MTB) calculated as 4MSB/(4MSB + 4MTB). **(C)** Allyl GSL accumulation average of the evaluated genotypes. The bar chart represents the mean and standard deviation. Each genotype within each treatment has a minimum of 60 independent plants measured. These plants were grown in a randomized block design with six individuals per genotype per treatment and 10 separate independent experiments. Means with the same letter show if the genotype's response to the treatment was statistically similar to Col-0 (a) or different from Col-0 (b) at *P* ≤ 0.05 from the two-way ANOVA analysis (Table S8).

To directly test whether alterations in the major genes of the GSL biosynthetic pathway controlling GSL structure and accumulation may play a role in the plant response to exogenous allyl treatment, we compared the response of seven GSL mutants to exogenous allyl GSL application (Tables S7, S8). This analysis showed that the genes controlling endogenous GSL accumulation and structure also influence how the plants biomass responds to the application of allyl GSL. The *gsm1* mutant in MAM1 at the *GS-Elong* locus abolished the biomass response of Col-0 to exogenous allyl GSL (Figure 8A). The introduction of a functional AOP2 enzyme into Col-0, a natural knockout, significantly decreased control plant biomass as if the plant was now responding to the endogenous allyl GSL. Interestingly, this line had a small but significant positive response to exogenous allyl GSL suggesting that the introduction of AOP2 and/or endogenous allyl GSL synthesis can shift Col-0 from having a negative response to exogenous allyl GSL to having a positive effect on biomass accumulation (Figure 8A). The *myb28/myb29* double knockout was similar to *gsm1* in that it abolished the Col-0 biomass response to exogenous allyl GSL treatment (Figure 8A).

In addition to altering biomass, the GSL mutants also identified differential GSL responses to exogenous allyl GSL in comparison to the Col-0 parent. Col-0 responds to exogenous allyl GSL by altering the oxidation status of the 4C GSL away from the 4MTB and toward the 4MSB (Figure 8B). Genotypes that abolished the plant biomass response to exogenous allyl GSL also tended to change internal GSL accumulation in response to the treatment. The altered response of these genotypes was not due to differences in allyl accumulation, suggesting that these effects are not simply via altered GSL metabolism (Figure 8C). Thus, known genes within the aliphatic GSL pathway can either abolish or invert both the plant biomass and GSL response of Col-0 to exogenous allyl GSL application.

## Discussion

Stabilizing dynamic response systems such as plant responses to biotic attackers typically requires some level of feedback wherein the response output modulates the upstream system. There is a growing body of knowledge regarding the vast suite of receptors present in the plant that measure diverse aspects of the biotic environment, including bacterial, fungal, insect, plant, and almost any form of antagonistic organism (Dangl and Jones, 2001; Jones and Dangl, 2006; Gouhier-Darimont et al., 2013). The perceived signals then feed into a downstream regulatory system that is often considered to involve key defense signaling hormones, including salicylic acid and JA-ILE, among others (Glazebrook et al., 2003; Glazebrook, 2005; Jung et al., 2009). However, plant defense outputs like secondary metabolites are not routinely tested for the ability to modulate these systems. In our study, we show that exogenous allyl GSL or some other component derived from allyl GSL can modulate both plant biomass and defense metabolite accumulation in *A. thaliana*. This effect is naturally variable and dependent upon the environmental conditions in which the plant is grown. Thus, it appears that, at the very least, allyl GSL and potentially other defense metabolites have the capacities to modulate plant biomass and defense outputs in the plant.

### Endogenous role of exogenous GSL

A number of lines of evidence support the idea that our application of exogenous allyl GSL is identifying an endogenous response. First, previous studies confirmed on the AOP2 gene have linked the level of endogenous allyl GSL to alterations in growth and development that were confirmed in with the application of exogenous allyl GSL (Kerwin et al., 2011; Züst et al., 2011). In our studies, applying 50 μM allyl GSL, fed to the roots led to altered plant biomass while accumulating to less than 5% of the endogenous pool, suggesting that we have not flooded the system (Figure 3). This suggests the plant biomass and GSL respond to a level of GSL that are within the endogenous range.

The application of 4MSB and allyl GSL produced different plant biomass responses in the same genotype under the same environment (Figure 2). This suggests that the effect is not caused by the common sulfate/thioglucose structure of these compounds but is instead related to the side-chain of the compounds. This could be caused by the intact GSL metabolite or potentially by compounds derived from the intact GSL. One potential mechanism is the activation/degradation of the GSLs by the myrosinases that in the indolic GSLs is known to affect their potential signaling roles (Bednarek et al., 2009; Clay et al., 2009). The myrosinases and associated nitrile specifier proteins can cause the GSLs to be converted into isothiocyanates, oxazolidine-2-thiones, nitriles, epithionitriles, and thiocyanates (Lambrix et al., 2001; Burow et al., 2006). For the two tested GSLs (allyl and 4MSB) myrosinases to treat them identically with release of the sugar and sulfate (Halkier and Gershenzon, 2006). In the leaves of Arabidopsis, the allyl GSL can be converted to the epithionitrile while the 4MSB can be converted to the simple nitrile depending upon the genotype (Lambrix et al., 2001; Wentzell and Kliebenstein, 2008). However, in the roots, there is no epithionitrile formation and allyl and 4MSB are equally converted to the same structural derivatives (nitrile and isothiocyanate) in all tested accessions (Wentzell and Kliebenstein, 2008). This suggests that if the difference between the two compounds is related to epithionitrile vs. nitrile formation that this would have to occur within the leaf after transport from the root. In some accessions, both the allyl and 4MSB GSLs are converted into potentially toxic isothiocyanates upon tissue disruption. However, some accessions that create predominantly the isothiocyanate, like Col-0, show a positive biomass response to allyl GSL which would suggest that this is not a toxin mediated effect. Arguing further against the idea that myrosinase mediate activation is the key player is the fact that endogenous myrosinase activation of aliphatic GSLs requires tissue disruption and our assay is being conducted on intact and undisturbed plants wherein there should be no co-occurrence of myrosinase and exogenous GSL. Together, this suggests that 4MSB and allyl GSL cause different responses but the specific active components mediating this response and the active site(s) in which this response is stimulated need to be elucidated by further experiments.

Interestingly, foliar analysis of the treated seedlings showed that the exogenous allyl GSL is absorbed by the roots and transported into the leaves. This indicates that allyl GSL must move into the root vasculature, at which point it would intermingle with the endogenously transported GSL (Nour-Eldin et al., 2012; Andersen et al., 2013). The major GSL transporters in the vasculature do not distinguish between different GSL structures (Nour-Eldin et al., 2012; Andersen et al., 2013). Thus, the exogenously applied allyl GSL will follow the same transport and accumulation routes as the endogenous GSL (Nour-Eldin et al., 2012; Andersen et al., 2013). In combination, these points support the idea that the Arabidopsis responses to exogenous allyl GSL treatment we observe go through the GSL metabolite, and not only AOP2 RNA, being the result of an endogenous process. Further work to identify the mechanisms behind exogenous allyl GSL induced responses is required to prove this hypothesis.

### Sucrose dependency of allyl GSL effects

The ability of exogenous allyl GSL to accumulate within Arabidopsis and to also alter plant biomass was dependent upon sucrose concentration. One possibility is that sucrose is merely altering the energy status of the plant and the sucrose impact on allyl GSL effects is simply indirect. An alternative idea arises from recent work that is showing that sucrose can function as a signaling molecule that influences a large array of developmental processes through interacting with diverse pathways including cell cycle, cell wall signaling, hormonal regulation, vacuolar transporters, programmed cell death pathways, ribosomal biogenesis and the regulation of translation (Ruan, 2012; Tognetti et al., 2013). This raises the possibility that allyl GSL effects somehow intersect with the sucrose signaling pathways. Supporting this possibility is the observation that some accessions show opposing exogenous GSL responses depending upon sucrose which indicates that there is some cross-talk between intracellular pathways elicited by sucrose and those endogenous signals related to exogenous GSL application. Interestingly, our results suggest that sucrose responses are naturally variable within Arabidopsis and that it may be possible to find additional players within this response network using quantitative genetics. Future work will have to identify the mechanistic basis of how allyl GSL alters biomass and defense compound accumulation to assess how cross-talk may be occurring.

### Response of plant biomass and GSL traits to exogenous allyl GSL treatment are linked

Feeding exogenous allyl GSL to 96 Arabidopsis accessions identified a wide range of genetic diversity in the plant biomass and GSL responses (Figure 5). Utilizing this natural variation, we were able to develop a model in which we could link the variation in GSL responses to the plant biomass responses (Figure 7). This model indicated a negative correlation between plant biomass response and the ratio of methylthioalkyl/methylsulfinylalkyl GSL and positive correlation between plant biomass response and total aliphatic GSL accumulation (Table S6). These results suggested that accessions that increase their biomass in response to exogenous allyl GSL application also increase their GSL content per unit biomass. Additionally, there is a structural specificity to this relationship in that increase in methylthioalkyl GSL is negatively associated with plant biomass.

In addition, the model indicated a correlation between multiple aliphatic GSLs and the plant biomass accumulation response. To test if endogenous GSL accumulation alters biomass accumulation, we utilized a T-DNA insertion line that abolishes the enzyme responsible for various chain elongated aliphatic GSLs accumulation, MAM1 (*gsm1*). This mutant abolished the response to exogenous allyl GSL, suggesting that MAM1 plays a role in this response in *A. thaliana* (Figure 8; Haughn et al., 1991). Another mutant line, *myb28/myb29* double knockout that dramatically reduces the accumulation of most aliphatic GSLs also did not respond to exogenous allyl GSL (Sønderby et al., 2007, 2010). Both the natural variation in endogenous allyl GSL levels as well as the feeding of exogenous allyl GSL suggested that the total level of allyl GSL impacts plant biomass (Figures 1, 5). In agreement with this, the introduction of a functional *AOP2* gene into Col-0 accession, a natural *AOP2* knockout, led to a significant reduction in biomass that was also associated with a loss of responsiveness to exogenous allyl GSL (Figure 8). Thus, the combination of data strongly supports the hypothesis that the response to exogenous allyl GSL mimics the biomass response to endogenous allyl GSL. Thus, GSL biosynthetic and regulatory genes can either abolish or invert the biomass response in Col-0 to exogenous allyl GSL suggesting that endogenous perturbations in GSL structure and accumulation affect this response. This interplay of exogenous allyl GSL with endogenous GSL further suggests that this response is an in planta component of how the plant connects defense to physiology.

In conclusion, Arabidopsis can respond to allyl GSL by modifying its biomass accumulation and its endogenous GSL pool dependent upon the environment and endogenous GSL genetic variation. This process utilizes the endogenous GSL biosynthetic pathway, key developmental regulatory genes and probably a set of uncharacterized genes. Further genome-wide association studies will help to elucidate the regulatory network and candidate genes controlling plant biomass response variation to exogenous allyl GSL.

## Author contributions

MF, MB, and DK conceived and designed the experiments. MF, HC, BL, CL, and RK conducted the plant work. MF, BJ, JC, and DK did the statistical analyses. MF, DK interpreted the data and wrote the paper.

## Funding

This work was funded by a Marie Curie International Outgoing Fellowship within the 7th European Community Framework Programme (PIOF-GA-2010-275286), the NSF DBI grant 820580 to DK, the NSF MCB grant 1330337 to DK, the USDA National Institute of Food and Agriculture, Hatch project number CA-D-PLS-7033-H to DK and by the Danish National Research Foundation (DNRF99) grant to DK and MB.

### Conflict of interest statement

The authors declare that the research was conducted in the absence of any commercial or financial relationships that could be construed as a potential conflict of interest.
